# Psychiatry in the age of AI: transforming theory, practice, and medical education

**DOI:** 10.3389/fpubh.2025.1660448

**Published:** 2025-09-29

**Authors:** Hongyan Zheng, Xizhe Zhang

**Affiliations:** ^1^Kangda College, Nanjing Medical University, Lianyungang, China; ^2^Early Intervention Unit, Department of Psychiatry, The Affiliated Brain Hospital of Nanjing Medical University, Nanjing, Jiangsu, China; ^3^School of Biomedical Engineering and Informatics, Nanjing Medical University, Nanjing, China

**Keywords:** artificial intelligence, psychiatry, medical education, ethics, diagnostic classification, algorithmic bias

## Abstract

Mental disorders constitute an urgent and escalating global public-health concern. Recent advances in artificial intelligence (AI) have begun to transform both psychiatric theory and clinical practice, generating unprecedented opportunities for precision diagnosis, mechanistic insight and personalized intervention. Here, we present a narrative review that examines the current landscape of AI-enhanced psychiatry, evaluates AI's capacity to refine diagnostic nosology, elucidate etiological mechanisms, formalize diagnostic criteria and optimize treatment strategies, and delineates the concomitant ethical, legal and social challenges–most notably those arising from data privacy, algorithmic bias and inequitable access to technological resources. In parallel, the review interrogates the implications of this technological inflection point for medical education. It argues that contemporary training programs must evolve through systematic curricular re-design, the incorporation of computational and data science competencies, the adoption of integrative pedagogical models that couple theoretical instruction with hands-on algorithmic practice, and the reinforcement of bioethical literacy. Only by embedding AI fluency within a robust framework of humanistic and professional values can the next generation of psychiatrists be equipped to harness algorithmic tools responsibly and to translate their outputs into clinically meaningful decisions.

## 1 Introduction

Mental disorders have become one of the most pressing global public health challenges. The World Health Organization (WHO) projects that, by 2030, mental illness will represent the leading contributor to the worldwide burden of disease (1). Complementary longitudinal analyses indicate that roughly one half of the global population is likely to experience at least one clinically diagnosable mental disorder before the age of seventy-five (2). Yet, despite the escalating demand for mental-health services, conventional psychiatry remains constrained by longstanding diagnostic and therapeutic limitations. Current classificatory systems—exemplified by the DSM and ICD—rely primarily on subjective symptomatology and lack objective biomarker support, whereas treatment selection is frequently guided by empirical trial-and-error, resulting in pronounced inter-individual variation in efficacy (3). This intrinsic “imprecision” contributes to misdiagnosis, under-diagnosis, and delayed intervention, underscoring the urgent need for transformative technological paradigms in psychiatric care.

Recent advances in artificial intelligence (AI) offer such a paradigm. Owing to its capacity for high-dimensional data integration and pattern discovery, AI has demonstrated promising potential to redefine disease classification, elucidate etiological mechanisms, refine diagnostic processes, and enable precision therapeutics (4–7). Machine-learning models trained on multimodal datasets—spanning neuroimaging (8, 9), electronic health records (EHRs) (10), wearable-sensor streams (11, 12), and social-media behavior (13)—can delineate biologically grounded subtypes of mental disorders and predict individual treatment response. Natural-language-processing (NLP) methods applied to unstructured clinical text further permit the extraction of latent symptom signals, enhancing early screening, risk stratification, and outcome forecasting (14–16). Nevertheless, most existing investigations are confined to small-scale or single-center cohorts, and unresolved issues of data privacy, algorithmic bias, and ethical accountability continue to hinder large-scale translation (17). Moreover, the epistemic implications of AI for foundational psychiatric theory remain insufficiently examined, and the pathways from data-driven discovery to routine clinical deployment require deeper elaboration.

The scholarly conversation has accelerated, with several recent reviews surveying digital mental health, generative AI, and implementation challenges (18–20). These contributions map opportunities and risks in apps, chatbots, and immersive technologies and call for standardization, equity safeguards, and stronger evidence pipelines. Yet most syntheses remain tool-centric or service-oriented and less often bridge mechanistic biomarkers with clinical workflow redesign and professional education. We complement and extend that literature by integrating three levels of analysis within a single framework: mechanistic and theoretical advances, clinical applications and service redesign, and pedagogical and governance implications for medical education.

**Figure 1:**
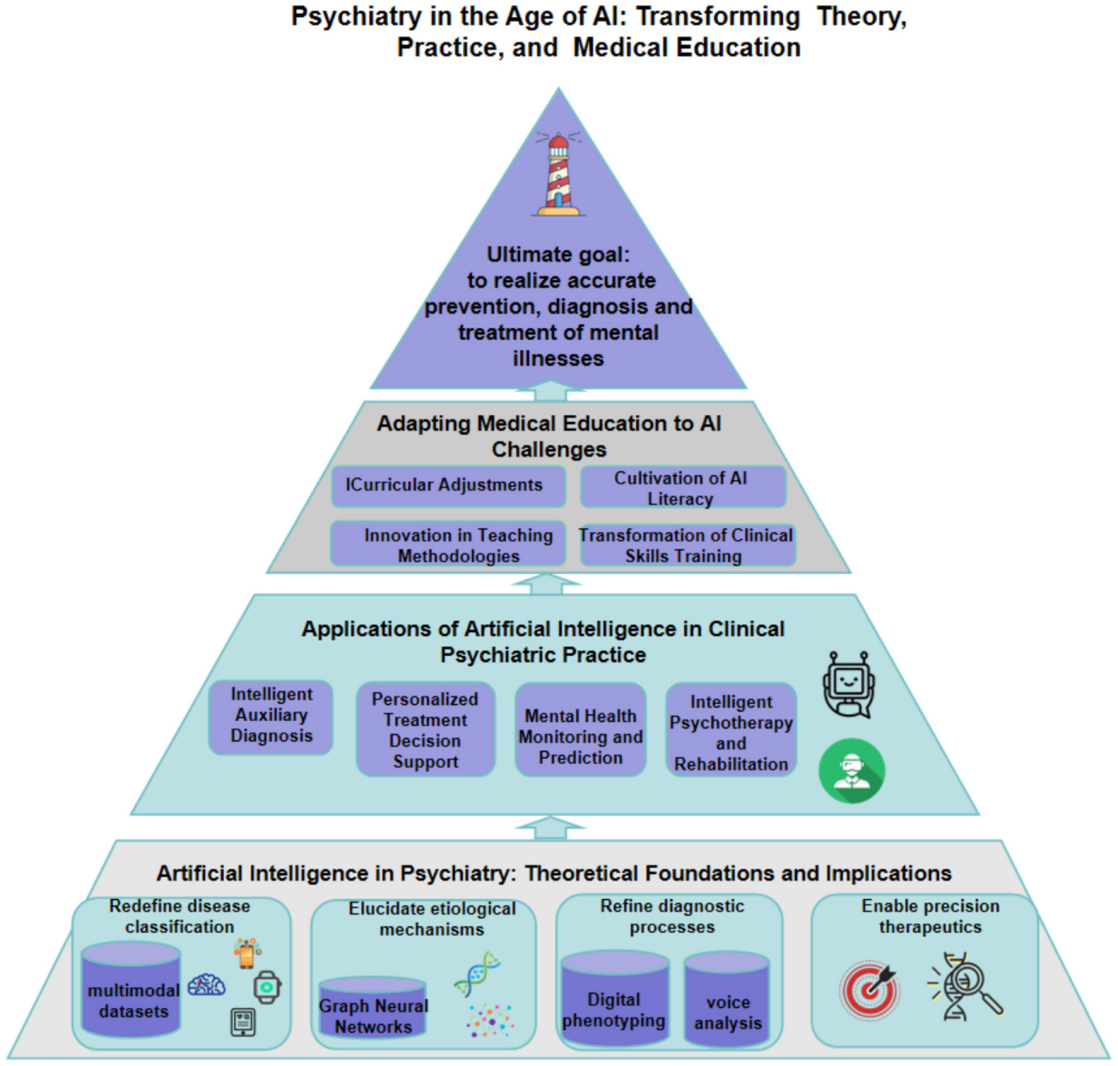
Overall structure of the paper.

In this narrative review, we first synthesize AI-mediated contributions to psychiatric theory across four domains: the reconstruction of nosologically systems, the elucidation of causal mechanisms, the objectification of diagnosis, and the individualization of treatment. Second, we survey the clinical progress of AI-augmented psychiatry in decision support, continuous health monitoring, and technology-assisted psychotherapy. Finally, we propose evidence-informed strategies for curriculum reform, advocating an integrative educational model that balances technical proficiency with ethical and empathic patient care. In delineating these dimensions, the article furnishes a conceptual roadmap for psychiatry in the AI era and offers practical guidance for optimizing the medical-education system. The structure of the whole paper is shown in Figure 1.

## 2 Methods

We employed a structured search to ensure breadth and transparency. We queried PubMed, Web of Science and IEEE Xplore for records published between January 1, 2005 and May 15, 2025, with no geographical restrictions. Searches combined controlled vocabulary and free-text terms spanning three conceptual blocks: psychiatry/mental health, artificial intelligence/machine learning (including deep learning, GNNs, and large language models), and application domains (diagnosis, treatment response, neuroimaging, EHRs, wearables, digital phenotyping, and medical education). Two authors independently screened titles/abstracts and full texts; discrepancies were resolved by consensus. We included human-participant empirical studies, methodological papers with clinical or neurobiological data, systematic reviews, and policy/curricular frameworks directly informing AI in psychiatry; purely technical reports without mental-health relevance, single-case reports, and animal-only studies were excluded. For pharmacotherapy response studies, drug-specific evidence was required to avoid unwarranted class-level generalization. For each included study, we extracted design, setting, sample, data modality, AI method, task, validation, and quantitative results where available. No meta-analysis was undertaken due to heterogeneity; findings were narratively synthesized with attention to effect sizes and external validity when reported.

## 3 Artificial intelligence in psychiatry: theoretical foundations and implications

Over the past decade artificial intelligence (AI) technologies—most notably machine learning and deep-learning algorithms—have progressed at an extraordinary pace. These models can autonomously extract multilevel features and discern complex patterns in large-scale datasets; in domains such as image recognition and natural-language processing they already equal or surpass human performance (21). Medicine has rapidly adopted this paradigm: by the close of 2023 the United States Food and Drug Administration had authorized almost 700 AI-enabled medical devices, and algorithmic decision aids are proliferating across diverse clinical specialties (22).

In the field of psychiatry, the introduction of AI holds significant promise for addressing long-standing challenges. Current psychiatric diagnosis primarily relies on classification systems such as the Diagnostic and Statistical Manual of Mental Disorders (DSM), depending on symptomatologic assessment and lacking objective biological markers. This subjective classification approach results in considerable diagnostic heterogeneity among patients, and clinical categories may not correspond to singular etiological mechanisms (23). Many mental disorders exhibit high rates of comorbidity and symptom overlap, blurring the boundaries between different diagnoses and thereby increasing the risk of misdiagnosis and underdiagnosis. Furthermore, the etiology of mental disorders is highly complex, involving an interplay of multiple factors including genetic, neurobiological, biochemical, and environmental influences, making it challenging for any single hypothesis to fully elucidate their pathogenesis. Significant inter-individual variability also exists in treatment response, with the same therapeutic modality yielding markedly different outcomes across patients. For example, in a recent multimodal deep graph learning study, sertraline response could be predicted from baseline brain network signatures (24); however, in routine care, first line antidepressant treatment achieves remission in only a minority of patients (e.g., ~28–33% in the STARD level 1 trial with citalopram), necessitating subsequent treatment steps for many (25). The diagnostic uncertainty and the trial-and-error nature of treatment underscore the imprecision dilemma within psychiatry. The strengths of AI technology in data mining, feature extraction, and pattern recognition offer novel tools to address this situation. Machine learning enables the discovery of subtle patterns from multimodal data—patterns often imperceptible to the human eye—thereby providing a foundation for the reclassification of mental disorders, etiological exploration, objective diagnosis, and precision treatment.

Overall, AI is progressively empowering psychiatric research and theoretical development, with the potential to transform traditional psychiatry by, for example, predicting disease trajectories, assisting in diagnostic and therapeutic decision-making, and enabling personalized intervention strategies (26).

### 3.1 Redefining the classification of mental disorders

Traditional classification systems for mental disorders (e.g., DSM and ICD) categorize complex psychiatric conditions into several classes based on clinical symptoms. However, the subjectivity inherent in these criteria frequently leads to “heterogeneity within diagnoses and homogeneity across diagnoses”: patients classified under the same diagnosis may exhibit vastly different symptom constellations, while conversely, patients with similar underlying etiologies might be assigned different labels under current standards. Consequently, clinical diagnostic categories may not accurately reflect the biological underpinnings of mental disorders (23).

With the ascent of data-driven methodologies, researchers have begun to leverage AI to reshape the boundaries of psychiatric classification. Machine learning models can integrate multimodal data (e.g., neuroimaging, genetics, neuropsychological assessments, and clinical rating scales) to automatically learn feature patterns from objective data, thereby proposing more granular or entirely novel classification schemes compared to traditional approaches (27). Such reclassification based on objective data holds promise for dissecting the heterogeneity of mental disorders: accumulating evidence suggests that subtypes identified through multidimensional data fusion can correspond to different disease trajectories and treatment responses, thereby enhancing the accuracy of clinical prediction (28–32).

Simultaneously, some studies have moved beyond traditional diagnostic frameworks by adopting transdiagnostic dimensional models (such as the RDoC framework proposed by the U.S. National Institute of Mental Health). These studies utilize AI to identify common pathological patterns across functional domains like cognition, affect, and arousal, aiming to elucidate the mechanisms underlying high comorbidity rates (23). Notably, multimodal data fusion imposes greater demands on sample size and algorithmic performance. Preliminary research indicates that multimodal AI models outperform single-modality models in discriminating between individuals with mental disorders and healthy controls, contributing to increased diagnostic objectivity (33, 34). Nevertheless, the current endeavor to redefine psychiatric classification using AI still faces considerable challenges. On one hand, the data-driven subtypes identified in various studies may not consistently replicate across different cohorts, indicating a need for improved reproducibility. On the other hand, the clinical significance of these algorithmically generated new categories remains unclear, and their limited interpretability restricts clinical application. Furthermore, achieving multimodal integration requires overcoming difficulties in data sharing and standardization, as well as persuading the clinical community to adopt new classification paradigms.

Despite these challenges, the AI-driven reconstruction of classification systems offers a novel perspective for psychiatric theory: it provides an opportunity to define mental disorders more precisely based on objective biological indicators rather than subjective symptoms, laying the groundwork for future updates to diagnostic manuals and personalized treatment.

### 3.2 Etiological hypotheses and mechanistic exploration

Elucidating the etiology and mechanisms of mental disorders has consistently been a core scientific challenge in psychiatry. Traditional research has often focused on single-level hypotheses, such as neurotransmitter imbalances, specific gene mutations, or structural abnormalities in brain regions. However, mental disorders are likely the result of multifactorial interactions, making them difficult to analyze using linear approaches. Artificial intelligence offers powerful tools for this endeavor: it can mine complex non-linear associations within high-dimensional data, thereby aiding in the generation and validation of novel etiological hypotheses.

Deep learning methods, such as Graph Neural Networks (GNNs), are increasingly being applied to the study of mechanisms in mental disorders. The brain is inherently a complex network, and GNNs can represent neuroimaging data as graph structures (where nodes represent brain regions and edges represent functional connectivity, etc.), thereby capturing intricate interaction patterns between different brain regions (35). Research indicates that GNNs possess unique advantages in characterizing brain functional architecture and can reveal network topological changes that are difficult to identify using traditional methods. In major depressive disorder, a multimodal deep graph learning study integrating resting state fMRI and EEG derived baseline brain network signatures that predicted differential outcomes to sertraline vs. placebo. Salient nodes for sertraline response included the inferior temporal gyrus and posterior cingulate cortex, whereas placebo response prominently involved the precuneus and supplementary motor area; cross modal consistent nodes included the superior temporal gyrus and posterior cingulate. The implicated connections spanned the frontoparietal control, dorsal/ventral attention, and limbic networks. Model performance reached mean *R*^2^ of approximately 0.24 (sertraline) and 0.20 (placebo), with best runs up to *R*^2^ ≈ 0.31 and *R*^2^ ≈ 0.28, respectively (permutation *P* < 0.001) (24).

Beyond brain network analysis, AI is also being employed to integrate data from genetics and large-scale electronic health records (EHRs) to discover novel combinations of pathogenic factors. Ensemble learning, by amalgamating predictions from multiple models, enhances the ability to identify weak signals. For example, a study on functional outcomes in schizophrenia inputted genetic polymorphism data into an ensemble algorithm. Through feature selection, it identified gene variant loci (e.g., G72 and MET gene polymorphisms) strongly associated with prognosis and validated that this ensemble model outperformed traditional statistical methods in predicting patients' quality of life and functional levels (36). These results suggest that the aforementioned genetic pathways may influence the long-term trajectory of the disorder, providing clues for in-depth research into the biological mechanisms of schizophrenia. Similarly, in disorders such as depression and bipolar disorder, machine learning combined with genetic and epigenetic data has identified several potential novel risk genes and molecular pathways. Furthermore, AI is being utilized to construct complex causal inference models and knowledge graphs, linking neurophysiology, environmental stressors, and behavioral manifestations to simulate the disease development process holistically. By conducting 'virtual experiments' within these models, researchers can test the strength of causal relationships among different factors, thereby providing quantitative support for etiological hypotheses.

Overall, artificial intelligence is facilitating a paradigm shift in psychiatry from experience-driven to data-driven approaches, and from univariate to multidimensional network perspectives. With the accumulation of more high-quality data and the emergence of more powerful algorithms, it is anticipated that we can more profoundly unravel the complex etiological networks of mental disorders, providing empirical evidence for theoretical models.

### 3.3 Diagnostic criteria and objective indicators

Due to the absence of visible biomarkers, psychiatric diagnosis has long relied on clinical symptomatology and patients' subjective reports, a model susceptible to the experience of the evaluator and the expressive capacity of the patient. To enhance diagnostic objectivity, researchers have begun to explore novel concepts and technologies such as digital phenotyping.

Digital phenotyping refers to the continuous quantification of an individual's behavioral and physiological states using objective data from personal digital devices (37). Because most people carry smartphones and wearables, passive sensing streams—such as accelerometer and gyroscope derived activity, GPS based mobility, and microphone derived prosodic features—and human–device interaction traces—such as communication frequency, social media use, and typing dynamics—offer high frequency correlates of mental state (11, 13, 37). Early platform work introduced a scalable, customizable smartphone research framework for data driven psychiatry, but it did not evaluate clinical efficacy and should not be taken as evidence that such tools already enable objective diagnosis or continuous monitoring in routine care (38). Likewise, the SEARCH cohort (39) is a school-based longitudinal study of child health that neither focused on digital phenotyping or AI in psychiatry nor employed personal electronic devices; its findings are not generalizable to psychiatric disorders. More recent overviews synthesize opportunities and limitations in AI-enabled digital mental health—including privacy, consent, reproducibility, and equity—and emphasize the need for stronger, externally validated evidence (3, 40, 41). Within this cautious frame, machine learning analyses of smartphone and wearable signals have related fluctuations in mobility, social interaction, and diurnal regularity to changes in depressive symptoms, pointing to a research pathway toward dynamic, objective monitoring (11).

Similarly, voice analysis has emerged as an important tool for digital phenotyping. Studies have found that patients with depression or anxiety may exhibit slower speech rates, monotonous intonation, and use more negative vocabulary (42). AI-driven speech processing models can quantitatively capture these subtle changes and have demonstrated high accuracy in identifying conditions such as depression, anxiety, and post-traumatic stress disorder (PTSD) (43–45). Reports indicate that acoustic and linguistic features extracted by deep learning frameworks can be used for the early diagnosis of mental disorders, with accuracy in some studies surpassing that of clinical interviews (46, 47).

More importantly, digital phenotyping paves the way for establishing objective quantitative assessment tools. Through continuous monitoring, clinicians can obtain information beyond the scope of outpatient interviews, such as a patient's sleep fluctuations or degree of social isolation over a week, thereby expanding diagnosis from a static, “snapshot” assessment to a dynamic portrayal of the patient's daily functioning (48). Some researchers propose integrating these digital indicators with existing diagnostic systems, for example, by introducing objective quantitative scales into the DSM diagnostic process or developing intelligent applications for auxiliary diagnosis, to achieve a fusion of subjective reports and objective data (42). This approach complements the “biopsychosocial” model, providing a fourth dimension of support for the diagnosis of mental disorders.

Of course, the genuine integration of digital phenotyping into clinical practice requires addressing numerous issues. Firstly, there are privacy and ethical considerations: continuous monitoring of personal behavior may infringe upon privacy, necessitating informed consent from patients and robust data security measures (42). Secondly, technical standards are crucial: different studies use varied sensor types and feature extraction methods, lacking uniform standards, and differences in digital behavior patterns across diverse populations and cultural backgrounds also require calibration. Finally, large-scale prospective studies are needed to validate the reliability and clinical utility of digital biomarkers to persuade clinicians to adopt these new indicators.

Despite the substantial challenges, digital phenotyping represents a future direction for the objectification of psychiatric diagnosis. With the increasing prevalence of mobile devices and advancements in AI analytical capabilities, it is anticipated that a system of objective indicators, complementary to traditional symptomatology, can be established, making the diagnostic criteria for mental disorders more scientific and comprehensive.

### 3.4 Treatment strategies and precision psychiatry

“Precision psychiatry” is a concept that has gained prominence in recent years, introducing the principles of precision medicine to the mental health field (49, 50). Its core objective is to tailor treatment regimens based on an individual's biological characteristics and pathological mechanisms, thereby enhancing therapeutic efficacy and reducing trial-and-error approaches. Artificial intelligence provides crucial support for achieving this vision: by learning from vast clinical and biological datasets, AI models can help predict a patient's response to a specific treatment, inform clinical decision-making, and accelerate the development of novel therapies.

In the domain of clinical decision support, machine learning algorithms have been utilized to construct treatment response prediction models. Traditionally, psychiatrists often engage in iterative trials of pharmacological and psychotherapeutic interventions to identify suitable treatments for patients. AI models, however, can leverage a patient's baseline multidimensional data (including symptom assessments, neuroimaging, physiological indicators, genetic information, etc.) to predict the probability of their response to specific medications or therapies (24, 36). For example, in patients with depression, models may predict their sensitivity to SSRIs based on brain functional connectivity patterns and genotypes, thereby guiding physicians in selecting medication or switching to cognitive behavioral therapy.

Such precise predictions hold immense value in major mental disorders, as they can shorten the duration of ineffective treatment trials, facilitate earlier implementation of effective interventions, and improve overall therapeutic outcomes. Research has confirmed that brain biomarkers in different patient subtypes are closely associated with treatment outcomes: variations in brain networks often determine the intensity of effect of antidepressants or antipsychotics on patients (24, 51, 52). AI can mine these relationships and translate them into actionable clinical tools.

This indicates that through AI analysis, it is possible to discern, prior to treatment initiation, which patients are more likely to benefit from a specific intervention, thereby realizing individualized treatment strategies akin to “prescribing the right drug for the right patient.”

## 4 Applications of artificial intelligence in clinical psychiatric practice

### 4.1 Intelligent auxiliary diagnosis

In clinical practice, AI is progressively assuming the role of a “second diagnostic opinion.” Intelligent diagnostic systems can integrate patients' symptom descriptions, medical histories, and auxiliary examination results to provide preliminary diagnostic suggestions or risk assessments. For example, Natural Language Processing (NLP) techniques can extract pertinent information from physicians' notes within electronic health records (EHRs) and, through deep learning models, convert unstructured text into structured diagnostic cues. A 2024 study developed a Transformer model that automatically extracts multi-year health data of patients from medical records to predict future disease risk, generate differential diagnosis lists, and propose medication recommendations (53). Although this model is still in the validation phase and not yet directly applicable for decision support, it demonstrates the potential of AI to automatically organize and analyze complex clinical information to assist in diagnosis.

Furthermore, some clinically-oriented AI systems have already been implemented. For instance, mental health assessment chatbots can collect symptom information in real-time through conversations with patients, conduct preliminary screenings for common conditions such as depression and anxiety, and flag cases requiring further evaluation. Such AI-driven screening tools can save clinicians' time, enabling them to focus their efforts on patients who require heightened attention (53). Additionally, to provide a clearer overview of AI applications in psychiatry, we have categorized AI applications according to different clinical domains and annotated their current development stages, as shown in Table 1.

**Table 1:** Summary of AI applications in psychiatry by clinical domain and their current evidence maturity level.

**Clinical domain**	**AI application**	**Current evidence maturity**	**Key research/examples**
Diagnostic classification	Multimodal data fusion for subtype identification	Validation phase	(24–30)
Etiological exploration	Graph neural networks for brain network analysis	Pilot phase	(33–36)
Objective diagnosis	Digital phenotyping via smartphones and wearables	Deployment phase	(38, 41, 48)
Treatment prediction	Machine learning models for antidepressant response prediction	Validation phase	(22, 37, 55)
Intelligent diagnostics	NLP-based extraction from EHRs for risk stratification	Pilot phase	(58, 59)
Decision support systems	Predictive models for personalized treatment selection	Validation phase	(61–63)
Continuous monitoring	Real-time behavioral tracking via wearables and mobile data	Deployment phase	(64, 66)
Psychotherapy assistance	AI chatbots and VR-based therapeutic interactions	Pilot/validation phase	(59, 67, 68)

Evidence maturity levels are categorized as follows:

— Pilot Phase: Proof-of-concept studies, small samples, single-center.

— Validation Phase: Replicated in independent cohorts, methodological rigor improved.

— Deployment Phase: Implemented in real-world settings, with ongoing efficacy and safety monitoring.

Overall, AI-assisted diagnostic systems contribute to enhancing the early detection rates and accuracy of mental disorders. Particularly in resource-limited settings and environments with a shortage of specialized professionals, they can serve as a beneficial supplementary tool.

### 4.2 Personalized treatment decision support

AI capabilities extend beyond “diagnosis” to assisting in “decision-making.” In clinical psychiatry, each patient's response to treatment varies considerably. By analyzing the treatment processes and outcomes of a large cohort of patients using machine learning models, key features influencing therapeutic efficacy can be identified, thereby providing reference suggestions for individual patients during clinical decision-making (33). For example, AI models can predict a patient's probability of responding to a specific antidepressant or antipsychotic medication based on their baseline characteristics (including genetics, polysomnographic physiological indicators, and previous treatment responses). If a model predicts a poor response to a first-line medication for a particular patient, physicians can adjust the treatment plan promptly based on this information, avoiding delays associated with ineffective treatment. Such decision support systems effectively internalize vast clinical experience into algorithms, assisting physicians in making evidence-driven decisions.

Literature indicates that AI has the potential to redefine treatment strategies for mental disorders, rendering them more precise (3). For instance, machine learning can optimize drug dosages and combinations, reducing the risk of side effects. Deep learning algorithms can also discern progress in cognitive behavioral therapy from subtle changes such as handwriting and language patterns, thereby prompting therapists to adjust intervention strategies. It is crucial to emphasize that AI provides recommendations rather than directives; the autonomy for clinical decision-making remains with the physician. However, research suggests that with AI assistance, the consistency and rationality of clinical decisions may improve, and physicians' efficiency in utilizing big data may also be enhanced (54).

In the future, with the maturation and application of AI decision support systems in psychiatry, physicians will be able to formulate individualized treatment plans with greater confidence, providing patients with “the right treatment for the right person at the right time.”

### 4.3 Mental health monitoring and prediction

Mental disorders frequently follow recurrent and chronic courses, making longitudinal assessment essential for prognosis and relapse prevention. AI-enabled personal sensing and digital phenotyping leverage high-frequency data from smartphones and wearables to derive behavioral and physiological proxies of mental state. Passive streams (e.g., accelerometer- and gyroscope-derived activity, GPS-based mobility, microphone-based prosodic features) and human–device interaction traces (e.g., communication patterns, social-media language, typing dynamics) have been associated with fluctuations in symptoms and functioning, offering near-continuous signals that complement clinic-based assessments rather than replace them (11, 13, 37). In parallel, wearable physiology has shown promise for individualized prediction in specific contexts; for example, heart-rate-variability features captured by consumer-grade devices, combined with machine learning, have been used to predict the efficacy of group cognitive-behavioral therapy (12). Within this cautious frame, AI models trained on smartphone and social-media data are being investigated for risk stratification of depressive symptom worsening and suicidal ideation; while early reports and study protocols highlight feasibility, robust external validation and governance safeguards remain prerequisites for clinical deployment (40, 55).

Research has demonstrated that AI can predict an individual's risk of depressive episodes or suicidal ideation with considerable accuracy by analyzing social media posts and data from wearable devices (40, 55). Once a model identifies risk signals, it can promptly issue alerts to the patient, their family members, or healthcare providers, enabling intervention before a crisis occurs. This AI-based mental health monitoring overcomes the reliance of traditional healthcare on in-person visits, providing an opportunity for “silent” conditions to be detected in a timely manner.

In summary, AI-driven monitoring and prediction are transitioning mental healthcare from episodic, clinic-based encounters to continuous, dynamic management, thereby enhancing the capacity to prevent relapse and deterioration.

### 4.4 Intelligent psychotherapy and rehabilitation

In addition to auxiliary diagnosis and monitoring, AI is also directly involved in psychological intervention practices, giving rise to novel therapeutic modalities. For instance, chatbot therapy and virtual reality (VR)-assisted therapy are two emerging directions in recent years. Chatbots (e.g., Woebot) utilize Natural Language Processing (NLP) techniques to engage in conversations with users, guiding them to apply techniques such as cognitive behavioral therapy (CBT) to regulate emotions. Although current chatbot conversations remain relatively superficial, studies have indicated their potential to alleviate mild to moderate symptoms of anxiety and depression, serving as a supplement to traditional treatments.

More advanced therapeutic explorations combine virtual reality with large language models (LLMs). In 2024, Spiegel et al. (56) developed an extended reality (XR) AI assistant that integrates VR scenarios with the GPT-4 model to provide precise mental health support for patients with depression and anxiety. Upon wearing VR equipment, the system simulates therapeutic scenarios, converses with the patient, and provides responses based on CBT, while an emotion analysis module adjusts the virtual therapist's expressions and tone (57). Preliminary results suggest that such AI therapeutic assistants are attractive to patients reluctant to engage with human therapists, offering a new alternative to traditional in-person counseling.

Furthermore, AI technology is also being employed to enhance the accessibility and equity of mental health services. A 2024 study introduced an AI-supported self-referral chatbot within the UK's National Health Service (NHS), resulting in a significant increase in the proportion of individuals from ethnic minority groups seeking mental health services through this system. Feedback indicated that the AI chatbot reduced the cultural and linguistic barriers perceived by these groups when seeking help, contributing to overcoming long-standing health service inequalities (58). Thus, the application of AI in psychotherapy and rehabilitation is evident not only at the level of technological innovation but also in improving patient experience and healthcare equity.

In the future, as generative AI becomes more adept at understanding and responding to human emotions, we may witness AI therapeutic assistants that are more “empathic,” further expanding the boundaries of clinical psychiatric practice.

## 5 Adapting medical education to AI challenges: curricular and training strategies

### 5.1 Curricular adjustments and cultivation of AI literacy

In response to the profound impact of AI on psychiatry, medical education must proactively reform training programs to ensure that future physicians are competent in technologically empowered clinical environments. However, it is reported that most medical school curricula have not yet systematically incorporated AI-related content, and many medical students and physicians lack fundamental knowledge of AI principles and applications. This deficiency may hinder their ability to fully utilize AI tools upon entering clinical practice (59).

To bridge this gap, the medical education community is advocating for the integration of AI course modules into existing curricula. Some scholars have proposed the development of a standardized core curriculum in “Medical Artificial Intelligence,” incorporating four key pillars—technological concepts, model validation, ethical guidelines, and outcome assessment—into medical student training (60). For example, a group of medical students published an article in 2023 proposing an AI teaching syllabus for global medical schools, aiming to cultivate students' competencies at three distinct levels: “AI tool users (consumers),” “AI clinical translators,” and “AI developers” (61). This recommendation reflects the need for medical education to impart AI-related knowledge in a tiered manner, tailored to students' diverse backgrounds. All future physicians should possess basic literacy in selecting and using appropriate AI tools. Students with an interest in data science could delve deeper into machine learning methodologies to act as a bridge between clinical practice and technology. A select few, proficient in both programming and medicine, could become developers and leaders of medical AI systems.

Regarding implementation, some institutions have begun exploring pathways to incorporate AI into their curricula. For instance, Harvard Medical School has pioneered the integration of generative AI content into its medical curriculum, recognizing that this technological revolution will profoundly alter the essential skills required of physicians, thus advocating for students to “learn to coexist with AI as early as possible”(62). For institutions with already saturated curricula, elective workshops or online modules can be adopted to teach AI knowledge, flexibly broadening students' perspectives (59).

To integrating AI into the curriculum in resource-limited settings, a feasible pathway is to stage competencies across cost-sensitive tiers. First, anchor practical instruction in an open-source, CPU-only stack—Python with scikit-learn, pandas and standard plotting libraries—using de-identified or synthetic datasets (e.g., *Synthea*-generated EHRs and publicly available fairness-audit corpora). Offline, self-contained notebooks should prioritize classical machine learning, calibration, error analysis and model critique before introducing deep learning. Second, introduce a small pool of shared mid-range GPUs or tightly capped cloud credits only to demonstrate concepts that truly benefit from acceleration, such as basic sequence modeling or clinical NLP; favor inference-only exercises with distilled models, small batch sizes and non-identifiable data to control costs and governance risk. Third, adopt a training-of-trainer approach to faculty development, providing reusable case bundles, solution keys, performance rubrics and objective assessments. Across all tiers, integrate data-governance and algorithmic-fairness exercises and make learning outcomes explicit so that students understand first principles, appropriate indications and limitations of AI. The pedagogical aim is to cultivate critical users of AI—neither uncritical adopters nor reflexive rejectors. In terms of workload, AI may reduce repetitive answering and grading but adds responsibilities in task design, oversight and integrity assurance; thus, its effect is best framed as a redistribution of effort rather than a guaranteed reduction.

### 5.2 Innovation in teaching methodologies

In addition to adjustments in curriculum content, teaching methodologies are also evolving with the integration of AI. AI technology can be leveraged to enhance the effectiveness of medical teaching and cultivate students' ability to adapt to future intelligent healthcare environments. A significant innovation is the introduction of intelligent tutoring and virtual simulation tools. For instance, AI-driven chatbot tutors have been employed to address challenging questions encountered by medical students post-lecture. By utilizing powerful NLP models, these chatbots can comprehend medical questions posed by students and retrieve information from vast medical knowledge bases to provide timely answers (63). Research indicates that such chatbot assistants, available 24/7, can offer personalized learning support to students, alleviate the burden on faculty for query resolution, and simultaneously help students consolidate knowledge and develop clinical decision-making skills through interactive dialogue.

However, it is also necessary to guide students in correctly perceiving the limitations of AI-assisted teaching. For example, current general-purpose chatbots may generate incorrect references or answers (the so-called “hallucination” phenomenon); therefore, students should use them as auxiliary tools under faculty guidance rather than as authoritative sources (63).

From an implementation standpoint, integrating AI typically redistributes faculty workload. Upfront effort increases for policy design, boundary setting, and academic-integrity oversight; once guardrails and assessment artifacts are established—such as requirements for process evidence, sampling-based verification of outputs, and brief viva-style defenses—chatbots can triage routine queries and enable more scalable formative feedback. The net time effect is context-dependent and should be evaluated within each course, while maintaining instructor accountability for high-stakes judgments.

Another important transformation is the advancement of virtual patients and simulation training. Traditional medical education often utilizes standardized patients (portrayed by actors) for students to practice history-taking and physical examinations. Now, large language models (LLMs) have made it possible to create realistic virtual patients. An educational team at Harvard Medical School developed a “Standardized Patient-Large Language Model (SP-LLM),” training an AI patient capable of interacting with students using institution-specific case data (64). Students can “communicate” with the virtual patient via text or voice, simulating the entire outpatient process from collecting medical history and conducting psychological assessments to proposing diagnostic and treatment plans. Commendably, these AI patients also provide feedback on student performance from the dual perspectives of “patient” and “assessor” after the simulation, including the thoroughness of clinical reasoning and communication skills (64). This innovative teaching method provides students with more opportunities for repeated practice in a risk-free environment, thereby enhancing their clinical skills.

Furthermore, AI is also being utilized for adaptive learning and assessment. Intelligent teaching systems can adjust the teaching pace and content according to students' mastery levels and even dynamically alter question difficulty during assessments to specifically reinforce weaker areas. These learner-centered pedagogical innovations fully embody the empowering role of AI–cultivating students' knowledge, skills, and competencies through personalized and intelligent means, thereby laying the foundation for their proficient application of AI in future clinical practice.

### 5.3 Transformation of clinical skills training

The era of artificial intelligence also compels medical educators to re-examine the priorities in clinical skills training. As AI assumes more prominent roles in diagnosis and decision-making, the skill set required of physicians will undergo adjustments. This is particularly true in the field of psychiatry. The value of psychiatrists largely resides in “soft skills” such as establishing therapeutic relationships with patients and conducting meticulous psychological assessments (60, 65). These capacities for humanistic care and communicative insight are core competencies that AI can hardly replace. Consequently, medical education needs to further reinforce the cultivation of humanistic competencies in medical students. For instance, during psychiatric clerkships and internships, greater emphasis should be placed on training students' communication skills, empathic abilities, and keen observation of patients' non-verbal behaviors (66). Even if AI can provide auxiliary diagnoses in the future, physicians will still need to understand patients' inner worlds and build bonds of trust through face-to-face interviews.

Furthermore, medical students should practice skills for collaborating with AI tools. This includes the ability to use and interpret AI-generated results in clinical contexts and to make final judgments after integrating AI suggestions with traditional diagnostic and therapeutic information. This essentially constitutes critical thinking training: students must learn neither to blindly follow AI nor to disregard it, but rather to use it as one reference for decision-making, adopting its outputs after their own analysis and verification (61, 62, 67). Some educational programs have begun to design case-based teaching, allowing students to use AI decision support tools in simulated scenarios and then discuss the reliability and ethical implications of AI conclusions, thereby cultivating their technological judgment.

Finally, medical education should also help students establish a correct professional identity—that future physicians must be both “technologically empowered” doctors proficient in AI and, more importantly, “true humanistic physicians” equipped with empathy and humanistic care. This requires a continuous emphasis on the values of medical benevolence throughout the teaching process, guiding students to reflect on the irreplaceable role of humans in healthcare. Some researchers have proposed a “Humanistic Medicine-Artificial Intelligence Integrated Education (HuMe-AINE)” framework, advocating for comprehensive reform in medical education through initiatives such as standardized AI competency training, full integration of AI tools into the curriculum, reinforcement of critical thinking exercises that merge technology and humanism, and reshaping the professional identity of physicians to encompass both technological and humanistic literacy (68).

In summary, medical education must embrace the challenges of AI with an open and forward-looking attitude. While imparting AI knowledge and skills to students, it must also solidify their humanistic foundation, cultivating a new generation of psychiatrists who can both utilize advanced technology and retain the warmth of a physician.

### 5.4 Regulatory frameworks and governance

The rapid advancement of AI in psychiatry has prompted international regulatory bodies to develop guidelines and frameworks to ensure these technologies are safe, effective, ethical, and equitable. Understanding these evolving regulatory landscapes is crucial for clinicians, researchers, and developers aiming to translate AI innovations into clinical practice.

World Health Organization (WHO): In 2021, the WHO published its guidance on Ethics and Governance of Artificial Intelligence for Health, which provides a comprehensive framework for addressing ethical challenges (69). The report outlines six core principles for the ethical use of AI in health: (1) protecting human autonomy; (2) promoting human wellbeing and safety and the public interest; (3) ensuring transparency, explainability, and intelligibility; (4) fostering responsibility and accountability; (5) ensuring inclusiveness and equity; and (6) promoting AI that is responsive and sustainable. For psychiatry specifically, the WHO's emphasis on equity is critical. It mandates that AI tools must be validated on diverse populations to avoid perpetuating global mental health disparities. Furthermore, the principle of protecting autonomy raises specific questions about informed consent for patients with conditions that may impair judgment, necessitating adaptable consent processes for the use of passive digital phenotyping and monitoring tools.

European Union Artificial Intelligence Act (EU AI Act): As the world's first comprehensive legal framework for AI, the EU AI Act adopts a risk-based approach, classifying AI systems into four categories: unacceptable risk, high risk, limited risk, and minimal risk (70). AI systems used in mental health are unequivocally classified as high-risk, falling under the product safety legislation for medical devices. This classification carries significant implications. Developers of AI-based diagnostic software or treatment decision-support systems for psychiatry must undergo a strict ex-ante (prior to market release) conformity assessment. This includes demonstrating robustness, accuracy, cybersecurity, and the provision of clear instructions for use. Furthermore, the Act mandates fundamental rights impact assessments and ensures human oversight, requiring that any AI-assisted psychiatric diagnosis must be validated or confirmed by a qualified human professional. The high-risk designation also demands rigorous post-market monitoring to identify and mitigate any emerging risks, such as algorithmic drift or newly discovered biases when deployed in real-world clinical settings.

U.S. Food and Drug Administration (AI/ML-Based Software as a Medical Device Action Plan): The FDA has taken a proactive but adaptive approach to regulating AI/ML-based medical software. Its central initiative is the development of a framework for predetermined change control plans (22). This acknowledges that AI models, unlike traditional medical devices, are designed to learn and improve over time. For psychiatry, this is particularly relevant for adaptive algorithms that personalize treatment recommendations based on continuous patient data input. A developer must pre-specify the types of modifications (SaMD Pre-Specifications, or SPS) and the associated algorithm change protocol (ACP) that will be used to retrain the model, ensuring that all updates are performed in a controlled and validated manner that maintains safety and efficacy. This approach aims to foster innovation while ensuring that “locked” algorithms (which do not change) and “adaptive” algorithms (which do change) are both appropriately monitored throughout their lifecycle. This is essential for ensuring that a therapy recommendation algorithm for depression does not evolve in an unpredictable or harmful way after widespread deployment.

The convergence of these frameworks highlights a global consensus on key tenets: the necessity of transparency, the imperative for robust clinical validation across diverse populations, the irreplaceable role of human oversight in psychiatric care, and the need for lifelong monitoring of AI systems. For the field of psychiatry, these regulations provide a crucial safeguard, ensuring that the pursuit of technological advancement is inextricably linked to the foundational principles of patient safety, equity, and professional accountability.

## 6 Ethical and societal challenges of AI applications and the role of medical education

### 6.1 Data privacy and security

The application of AI in psychiatry inevitably involves the collection and processing of highly sensitive personal mental health data. This raises significant privacy and security concerns. If patients' electronic health records, treatment histories, or even daily behavioral data are utilized for machine learning training and prediction, the methods for storing, sharing, and protecting such data become paramount ethical issues. Currently, due to concerns regarding data security and accountability, different institutions often adopt a cautious stance on data sharing. This hinders medical AI from accessing large-scale, multi-center datasets required to train high-precision models, thereby limiting model performance. Even when data are accessible, stringent de-identification and encryption measures must be ensured to prevent patient privacy breaches. Furthermore, if mental health data are improperly utilized (e.g., for commercial marketing or insurance risk assessment), it could cause harm to patients' rights.

Therefore, medical education should enhance data ethics training for future physicians, enabling them to fully recognize the importance of protecting patient privacy and to uphold their commitment to data security and patient informed consent in their professional practice (71). Only by ensuring that data are used in an ethical and secure manner can the value of AI in the mental health field be realized sustainably and responsibly.

### 6.2 Algorithmic bias and fairness

The performance and impartiality of AI models are highly dependent on the training data. If the training data are imbalanced with respect to race, gender, socioeconomic status, or other demographic factors, the model may inadvertently reflect these biases in its outputs, leading to systematically disadvantageous decisions for certain groups (17, 72). For example, if a model predicting suicide risk is primarily trained on data from Western populations, its accuracy may decrease or it may produce erroneous judgments when applied to patients from other cultural backgrounds, potentially delaying intervention or causing unnecessary distress.

Furthermore, psychiatric diagnosis itself is susceptible to preconceived notions; if AI learns from biased clinical records, it may reinforce existing prejudices.

To mitigate these issues, researchers are developing AI fairness auditing tools, such as Google's What-If Tool and IBM's AI Fairness 360, which can be used to detect performance disparities across different population subgroups and to facilitate adjustments (3, 54, 72). Among these, AI Fairness 360 implements bias detection metrics (e.g., statistical parity differences, differential impact, and equal opportunity differences) and three intervention series: pre-processing (e.g., reweighting, optimized pre-processing), in-processing (e.g., adversarial de-biasing), and post-processing (e.g., odds balancing and calibrated odds balancing). Through a workflow similar to “dataset → metric review → mitigation → re-review,” AI Fairness 360 is integrated into healthcare AI development and validation. Medical education should play a crucial role in this regard by cultivating in medical students an awareness to identify and question AI bias. When an AI system provides conclusions inconsistent with clinical intuition, physicians should be capable of inquiring whether this could be attributable to data or algorithmic bias. Through case-based teaching and ethical discussions, students can learn how to detect and report potential unfairness in AI systems and participate in their improvement. This will help ensure that future applications of medical AI do not compromise the interests of vulnerable populations.

It is noteworthy that, if designed and utilized appropriately, AI can also promote health equity—for example, by incorporating data from underserved regions into training to make models applicable to a broader population, or by leveraging AI-assisted decision-making to reduce the impact of human bias on diagnosis and treatment (72). Therefore, educating medical graduates about algorithmic fairness will equip them with the tools and responsibility to safeguard medical justice in their practice.

### 6.3 Transparency and accountability

Many AI models, particularly deep learning models, operate as “black boxes,” meaning it is difficult to explain to users the specific basis for a given decision. This is especially sensitive in psychiatric applications: if an AI suggests a patient has a high suicide risk but cannot elucidate which behaviors or features led to this conclusion, clinicians and patients may harbor doubts about its reliability. Opaque decision-making processes also pose challenges for accountability—if an AI's recommendation leads to misdiagnosis or mistreatment, should the algorithm developer, data provider, or the clinician using the AI be held responsible?

Therefore, enhancing the interpretability of AI decisions and establishing clear accountability frameworks are crucial aspects of AI ethics. Technologically, eXplainable Artificial Intelligence (XAI) is an emerging field aimed at providing human-understandable explanations for black-box models (73). In psychiatry, this might involve highlighting the symptoms or indicators that the AI deems most important, or providing references to similar cases, enabling clinicians to assess the rationality of AI recommendations. Some researchers advocate for prioritizing the use of interpretable models over purely black-box models for high-stakes clinical decision-making (74).

At the regulatory level, many countries are beginning to explore oversight and accountability mechanisms for medical AI, requiring AI systems used in clinical settings to meet certain standards and to clearly delineate the division of responsibilities among various parties in the event of adverse incidents (75). In this domain, medical education can facilitate ethical case discussions to encourage students to proactively consider dilemmas such as: what should be done when AI's opinion conflicts with a physician? How should errors resulting from reliance on AI be managed? By discussing these issues, students can cultivate the attitude and ability to appropriately supervise AI and be accountable to patients in their future professional practice.

In conclusion, enhancing transparency and clarifying accountability are not merely technical and legal issues but also essential professional competencies for future physicians.

### 6.4 Physician-patient relationship and humanistic care

Perhaps the most profound and subtle challenge for psychiatry lies in maintaining the centrality of humanistic care within a highly technologized healthcare environment. The efficacy of psychiatric treatment is largely dependent on the therapeutic alliance and empathic support—patients need to feel understood and cared for. With the increasing involvement of AI in diagnosis and treatment, concerns have been raised that this may weaken the emotional connection between physicians and patients, potentially rendering healthcare cold and impersonal. On one hand, if physicians become overly reliant on AI analysis, they might reduce face-to-face interaction time with patients or place insufficient emphasis on patients' subjective experiences. On the other hand, some patients may be resistant to AI intervention in mental health services, fearing being treated by a “machine” rather than cared for by a human. Furthermore, healthcare professionals themselves may experience anxiety about “replacement,” worrying that AI could devalue their meticulously honed clinical skills and affect their sense of professional meaning (76).

For medical educators, a crucial task is to strike a balance between technology and humanism, ensuring that graduating physicians are both proficient in AI and retain their human warmth. Specific strategies include: strengthening medical humanities education by integrating psychology, ethics, and communication skills training into the curriculum, enabling students to deeply recognize the indispensable role of human emotions and values in healthcare; fostering correct perspectives by emphasizing that AI is an auxiliary tool rather than a replacement for physicians, capable of processing information but unable to substitute for human understanding and compassion; and cultivating empathy through teaching methods such as patient role-playing and narrative medicine to reinforce students' perception of and compassion for patient experiences, thereby ensuring patient-centered care regardless of the extent of technological involvement in future healthcare (77–79).

As advocated by Jeste et al. (80) for AI to genuinely improve healthcare in the future, it must embody the concept of “Artificial Wisdom”—integrating the empathy and ethics of human intelligence to provide compassionate and ethical care. To this end, medical education must undertake the mission of cultivating students' ethical reasoning abilities and humanistic spirit, enabling them to both harness the “intelligence” of AI and uphold the “benevolence” of a physician.

## 7 Challenges and future perspectives

Despite the exciting prospects demonstrated by artificial intelligence in the field of psychiatry, its large-scale application still confronts numerous challenges, necessitating sustained efforts from the research, clinical, and educational communities.

### 7.1 Technical and clinical validation challenges

Currently, many AI models exhibit excellent performance on closed datasets; however, whether they can maintain this performance in complex real-world environments remains uncertain. Research has found that some machine learning models trained on data from large clinical trials experience a significant decline in performance when applied to independent, real-world clinical datasets (81). This indicates an urgent need to validate the external validity and generalizability of these models through studies involving broader populations and longer follow-up periods.

Furthermore, mental disorders are characterized by high heterogeneity and dynamism, making the development of AI models capable of capturing the full spectrum of disease and sensitive to individual differences a non-trivial task (29). Future research must strive to enhance model interpretability and robustness concurrently with improving accuracy. For instance, integrating interpretable models can help ensure clinician and patient trust in AI-generated conclusions, while training with multi-center data can improve model applicability across diverse populations (82).

### 7.2 Regulatory and standardization challenges

As AI tools progressively enter clinical practice, healthcare regulatory bodies need to establish corresponding standards and regulations to ensure their safe and effective use. This includes independent evaluation and approval of AI algorithms, as well as the establishment of continuous monitoring and updating mechanisms. Some countries have begun to issue provisional guidelines for medical AI, but globally harmonized industry standards have yet to be formulated. Legal frameworks for accountability and ethical guidelines also require further clarification to balance the promotion of innovation with risk mitigation (83). Collaborative dialogue among policymakers, technology developers, and clinical experts will be crucial in shaping a favorable environment for AI applications.

### 7.3 Future outlook

Looking ahead, artificial intelligence is poised for closer integration and synergy with psychiatry. Firstly, in the realm of scientific research, interdisciplinary collaboration will drive the advancement of computational psychiatry. The partnership between computer scientists and psychiatrists can lead to the development of models more attuned to clinical needs and can feed new data analysis findings back into disease theory research, accelerating the transition of mental disorders from conceptual classification to biomarker-based classification. Investment of funding and resources is also crucial—for instance, the U.S. National Institute of Mental Health (NIMH) has designated explainable AI as a priority funding area, and private technology companies are collaborating with mental health startups to translate research findings into clinical tools (3).

Secondly, concerning clinical applications, we may witness the emergence of AI systems that are more intelligent and possess “emotional intelligence.” Just as current voice assistants are continually becoming “smarter,” future psychiatric AI may, to some extent, simulate human emotional responses, becoming “machine colleagues” that genuinely understand patient emotions. Literature suggests that future AI will need to incorporate aspects of emotional intelligence, ethics, and empathy to be termed “Artificial Wisdom” rather than merely Artificial Intelligence (3, 41, 84). This implies the potential emergence of AI therapeutic assistants with personalized traits and emotional support robots; scenarios previously confined to science fiction that are gradually becoming feasible. Concurrently, the role of human physicians will also evolve: future psychiatrists will increasingly act as “supervisors” and “collaborators” with AI, combining their professional expertise with AI's computational advantages to serve patients collectively.

Finally, medical education will also be a process of continuous innovation. Curricular content must evolve with the times, promptly incorporating new AI developments and lessons learned. Teaching methodologies will fully leverage AI tools themselves, such as intelligent tutors providing personalized guidance to students and data analytics assisting in the evaluation of teaching effectiveness (85). A more profound change lies in the educational philosophy—emphasizing lifelong learning and interdisciplinary collaboration, encouraging medical students to continuously update their technological skills throughout their careers, and to collaborate with experts in fields such as engineering and ethics to solve complex problems. Only through such endeavors can the medical community ensure that AI technology is applied reasonably, effectively, and ethically in the field of mental health.

## 8 Conclusion

Artificial intelligence technology is reshaping the theory and practice of psychiatry at an unprecedented pace. From novel insights into disease classification and etiology at the research end, to intelligent diagnostics, personalized treatment, and real-time monitoring at the clinical end, AI has demonstrated immense potential for enhancing mental health services.

However, this transformation is accompanied by underlying concerns and challenges, reminding us that technological advancement must proceed in parallel with ethical reasoning. Medical education plays a crucial bridging role in this context: it must equip future psychiatrists with the “hard skills” of AI, while simultaneously reinforcing their humanistic literacy and ethical “soft power,” ensuring that physicians always prioritize patient wellbeing when collaborating with AI.

In essence, artificial intelligence is not an antagonist to human therapists but rather holds the promise of becoming a powerful partner for psychiatrists. Through a scientifically rigorous approach and prudent, comprehensive educational training, we can leverage the strengths of AI to compensate for human limitations, while guiding AI development with human wisdom in a direction beneficial to patients.

Looking forward, mental health services that integrate AI technology and humanistic care will become more efficient, precise, and compassionate, offering patients greater hope for recovery. Provided that we utilize artificial intelligence judiciously and adhere steadfastly to medical ethics, the future of psychiatry will undoubtedly usher in a new era of human-machine collaboration for co-creating health.
